# Language of Medical Instruction in Palestine: A Mixed Method Approach of Students' Perceptions

**DOI:** 10.1155/2022/8999025

**Published:** 2022-07-27

**Authors:** Oqab Jabali

**Affiliations:** Language Center Faculty of Humanities, An-Najah National University, Nablus, State of Palestine

## Abstract

This mixed method study explores medical students' perceptions and attitudes regarding the language(s) of medical instruction in two Palestinian universities. The researcher aimed to identify the way medical students look at the language of medical education as well as the merits and drawbacks of the language(s) used in medical instruction. A 25-item descriptive, online questionnaire was built to explore the way university students evaluate and perceive the medium of medical instruction at the Faculty of Medicine. To complement and inform the quantitative findings, fifty-five students from each university were randomly selected, and their responses to an open question about the merits and drawbacks of the language(s) were analyzed using MAXQDA. Of the too many medical students enrolled in the two universities, 604 completed and returned the survey, and 55 students were selected to interpret their open responses qualitatively. The study findings suggest that the students are divided into two camps concerning what the language of medical instruction should be; some prefer Arabic, their mother tongue, while the others showed no reservations about using the medical academic vocabulary in English. Some statistically significant differences were found when some demographic variables, i.e., gender, specific major, and year of study, interact. Finally, study respondents highlighted several issues which the researcher sorted into advantages and disadvantages for each language. There is a considerable discrepancy in the choice of the language of medical instruction at the Faculty of Medicine. Each language has its pros and cons; consequently, a mixture of a mother and a foreign language (e.g., English) could be a suitable compromise in a country like Palestine.

## 1. Introduction

There is no doubt that all countries have educational systems that are set to achieve their objectives and meet their citizens' aspirations. Traditionally, almost all countries have decided to prioritize the national language to be the language of instruction at all education levels, mainly, elementary schooling. However, over the past few decades, a rapid movement toward the incorporation of multilingual education has arisen in the Arab world.

The language of instruction is deemed significant for quality education; therefore, most countries strive to construct an educational system that may compete with other rivaling systems and positively impact the population's social, cultural, economic, and on top of all, scientific aspects. Among the most significant key component of successful educational systems is the medium of instruction, i.e., language, in which schooling and training are carried out [1].

People in the Arab world use Arabic for all traditional, everyday contextual discourse needs and interactions. It is the language of instruction at schools, and the majority of Arabs use it in healthcare centers and other medical institutions. Among Arabic-speaking countries, Syria may be the only one to have systematically adopted Arabic-based medical curricula for more than a century [2]. A college of medicine for Arabs opened in Damascus in 1919 [3]. Damascus University's experience with teaching medical sciences in Arabic is pioneering because students who studied medical sciences in Arabic language were not handicapped by the language, despite the fact that many individuals have demanded the teaching of medical sciences in English to keep Syrian students up-to-date on medical innovations [4]. Using students' native language has a significant positive impact on them; it increases learning and promotes participation [5]; it also helps students get rid of the burden of linguistic dualism [6]. Teaching students using their mother tongues facilitates topic acquisition and utilizes the optimum potential of the students [7]. The mother tongue should be employed in certain circumstances including, but not limited to, “explaining terminology and difficult content, repairing breakdowns, and encouraging student interaction, feedback, and participation” ([8], p. 16). It could be utilized in times of communication breakdown [9, 10] while giving instructions or telling jokes [11] if students' level of English is low [12, 13] and if the instructor does not speak English at all or the course content is challenging and, thus, entails using the mother tongue [13–16].

In fact, Arabic was one of the first lingua franca in history, especially during the golden age of the Arab and Muslim civilizations. A large bulk of literature has proved the advantageous aspect of teaching modern sciences including medicine in the students' mother tongue [2, 7, 17–19]. However, English has become the language of instruction in many fields of higher education such as biomedical sciences and engineering [12] in most Arab countries including Palestine. This tendency has resulted in a controversy about the pros and cons of teaching medicine in English.

There are hundreds of medical schools and institutions in the Arab world. Many of them currently teach medicine in English [7, 20, 21]. Nevertheless, the reason for using English as a language of medical instruction is centered around the fact that English is the first international language and that academic information, science, and technology are mostly expressed in English [22, 23] and that the official language of notable journals and international gatherings is English [24, 25].

Opponents of using English believe that medical students and doctors are very likely to face many difficulties. For instance, they get exposed to unfamiliar curricula which force them to keep thinking about grades and acquiring new terms [26]. Medical students may academically perform poorly [27–29] and have difficulty communicating with patients upon graduation [6, 30–35]. Furthermore, students' memories do not function properly [36]; students' brains might not process the input presented in a second language the way they do when mother tongues are used [37]. As stated by Breeze and Roothooft [8] (p. 15), sometimes “lecturing in English is still a challenge …, particularly when international students are a small minority.” In the two Palestinian universities, there are no international students although there are many Israeli-Arab students whose mother tongue is Arabic; they receive education in Arabic or Hebrew, but not in English.

The language of instruction for teaching medicine at the Faculty of Medicine and Health Sciences at all Palestinian universities is English even though the students' mother tongue is Arabic which is also the language of teaching at all levels of school education. All medical students, irrespective of their specific major at the faculty, are supposed to study at least two basic courses in the English language; one of them is English for Medical Purposes. It is worth mentioning that enrolled students come from different locations and have different linguistic levels based on regional and educational backgrounds. The researcher, who is an instructor of English as a Foreign Language, has noticed big gaps among students enrolled at the Faculty of Medicine mainly in the English for Medical Purposes course. There might be different reasons for this huge discrepancy among students' linguistic capabilities that may impact their performance positively and negatively and affect educational goals and objectives as well as the instructional strategies used by teachers.

This study intends to explore the perspectives of medical students on the choice between Arabic and English as the language for medical instruction in two Palestinian universities. It will highlight the advantages and disadvantages of the two languages from a pure students' perspective and eventually suggest the best language for teaching medicine. The researcher intends to answer the following questions: (1) Are there statistically significant differences among study participants concerning their favorite language of instruction at the faculty that may be attributed to the various demographic variables (e.g., gender, year of study, and specific major)? (2) What are the perspectives of medical students on the language of instruction in the Faculty of Medicine at the two intended universities?

## 2. Materials and Methods

### 2.1. Setting

This study involved all medical students enrolled at the Faculty of Medicine and Health Sciences at two Palestinian universities located in the West Bank during the 2019/2020 academic year. These students come from different locations and have different perspectives and ambitions. Although they belong to the same nationality, i.e., Palestinians, they received basic education in different locations: West Bank, Green Line areas inside Israel, and foreign countries mainly the Gulf States such as Saudi Arabia and the United Arab Emirates. Most of them received school education in Modern Standard Arabic; very few of them (mostly the rich) were taught using English based on their economic status and being in the Gulf States. Israeli-Arab students received school education in Arabic and Hebrew. English is, in fact, among the school topics included in the curricula.

### 2.2. Participants

The population of this study included all university students who were enrolled at the Faculty of Medicine and Health Sciences at two Palestinian universities A and B. In An-Najah National University in Nablus, Palestine (university A), more than 3500 students were enrolled in the Faculty of Medicine while Al-Quds University, Jerusalem, Palestine (university B) included approximately 1600 medical students. It is worth noting that the Faculty of Medicine at both universities includes five departments: Applied and Allied Medical Sciences, Nursing and Midwifery, Medicine, and Pharmacy. The study programs last between four and six years. Typically, the Pharmacy study program lasts for five years; the Medicine program lasts for six years, and the other remaining programs last for four years.

The survey respondents, who were chosen randomly as the questionnaire was distributed online, come from different socioeconomic and linguistic backgrounds. They represent diverse socioeconomic backgrounds and conditions; yet, those who choose to study medicine mostly come from high social classes because studying medicine costs much more money as compared to other specialties mainly those that last for four years. A large number of those who study medicine come from either Arabs living in Israel or those whose families live or work in Gulf State countries due to financial issues. Moreover, the respondents vary in terms of linguistic as well as educational potentials and capabilities; medical students are mostly better than the others in the faculty especially at English let alone other academic subjects [38]. In both universities, admission depends on students' exit exams and their ultimate high school GPA. The minimum GPA at university A is 4 or 95 for Medicine, 3.3 or 85 for Pharmacy, and 2 or 70 for the other programs while it is 90, 80, and 70, respectively, at university B. The respondents also come from the two universities, represent the five departments, and have different academic levels in the sense that there are freshmen, sophomores, juniors, seniors (fourth-year students), and super seniors (fifth- or sixth-year students). Furthermore, the respondents include males and females, and their ages range from 18 to 25. It is worth mentioning that these students were taught in Arabic, to a large extent, and Hebrew, to some extent, at the school level. Very few students were taught using English at schools [38].

### 2.3. Materials

To carry out the current mixed method study, ethical approval was obtained from the two university vice presidents of academic affairs, the deans of scientific research departments, and the research ethics committees. Quantitatively, a descriptive online questionnaire was built to explore the way medical students perceive the language of instruction in medical education. As the researcher was interested in examining relevant information efficiently and logically, the medium of instruction in the medical field is conceived comprehensively to include any possible reactions, attitudes, or comments that might be of any relevance to medical education at the university level.

Eighty declarative sentence items that relate to students' reactions, attitudes, or comments that might be of any relevance to medical education were built; a large bulk of these sentence items relate to the advantages and disadvantages of the two languages (i.e., Arabic and English) used at both universities as a means of instruction. Then, they were sent to 10 arbitrators who have adequate knowledge in the field; all of them hold Ph.D. degrees and work as faculty members; they have adequate experience in research writing and reviewing; some of them serve as journal editors or members of an editing board. To evaluate the survey, the arbitrator adopted the weighted average method because the survey consisted of subsections and some of these subsections have higher significance than others [38]. Their scores were collected, and the correlation between each sentence item and the total was calculated to find out how the scores of the items are related; the items whose correlation was less than 0.6 were deleted; then, the mean scores for the first and last quartiles were calculated for rating the items by the arbitrators. *t*-test of two independent samples between the means of the two quartiles for each item was calculated, and the sentence items for which the difference was not statistically significant were deleted simply because the researcher was interested in the items that are distinguished by high discrimination. In the end, 32 high discrimination items that were of high relevance and correlation to the medical language of instruction were retained.

To ensure the questionnaire validity, factorial validity was calculated using the Kaiser-Meyer Olkin test; it was 0.0955 which ensured that the items were suitable for the exploratory factor analysis. To exclude orthogonal items, the principal component method and oblimin rotation were used; consequently, items whose communality degree or factor loading was less than 0.3 were excluded. In total, 25 items were retained. A five-point Likert scale, with strongly agree (5), agree (4), uncertain (3), do not agree (2), and strongly disagree (1), has been used to measure the frequency of the 25 items.

To ensure the reliability of the questionnaire, Cronbach's alpha was calculated. Cronbach's alpha coefficient was 0.946 for the total 25 items. The alpha value was higher than 0.7; this shows the questionnaire is reliable.

### 2.4. Data Collection and Data Analysis

Eventually, the questionnaire was posted to medical students online; it was written in students' mother tongue, i.e., Arabic, to ensure that all students understand the survey items fully and adequately and, consequently, respond appropriately without falling under the pressure of interpreting the items if they were written in a different language let it be English or Hebrew, for example (Supplementary File (available here)). 604 survey instruments were completed and returned for analysis. Data collection was carried out during the first semester of the academic year 2019/2020.

Quantitatively, data were normally distributed and were analyzed using descriptive statistics; factorial ANOVA was used to calculate the mean differences between demographic element scores using SPSS version 26. The principal study tools also required students to provide information about their attitudes, opinions, and perspectives towards the language of instruction in medical education, and the advantages and disadvantages of the language used in the universities to complement and inform the quantitative findings by providing valuable data that help understand students' attitudes towards it. Qualitatively, MAXQDA was used by the researcher himself to calculate frequencies, percentages, and students' responses, opinions, and behaviors on the open question. MAXQDA is a software program designed for computer-assisted qualitative method data and text analysis; it offers tools for the organization and analysis of qualitative data especially those obtained as texts to attain valuable explanation and comprehensive understanding or interpretation of a phenomenon or a tendency [38].

Associations were tested at a 95% significance level (*P* < 0.05). Fifty-five students from each university were randomly selected after administering the questionnaire to ensure that these students include further attitudes, reactions, perspectives, advantages, or disadvantages related to the medium of medical instruction applied at the two universities that the researcher may have missed in the survey items, on the one hand, and give the students the chance to express themselves freely and frankly, on the other hand.

## 3. Results

### 3.1. Quantitative Results

#### 3.1.1. Demographic Characteristics and Perception of the Medium of Medical Instruction

To answer the first question, 604 students studying medicine at university A (*n* = 302) and university B (*n* = 302) responded to the questionnaire. Descriptive statistics of students' responses were calculated based on the demographic variables, i.e., gender, year of study, and specific major.

Female respondents (200 at university A and 210 at university B) outnumbered their male counterparts in both universities (102 at university A and 92 at university B) because more female students join universities rather than male ones; it is a common phenomenon in Palestine as males prefer to go to the labor market than spend 4-6 years of study and wait for job opportunities which are very rare in an occupied country. However, gender evaluation of using the two languages as means of medical instruction was high and nearly equal.

Another crucial variable was taken into consideration related to students' field of study or specific major. With a closer look at the normal distribution of students among departments, one could easily find that the largest number of respondents came from the Department of Pharmacy and the Department of Nursing and Midwifery at both universities. Each department was represented by 114 students in university A, while 120 respondents came from Pharmacy and 122 came from Nursing and Midwifery in university B. In fact, the tuition fees in these two majors are noticeable less than in Medicine and Applied and Allied Medical Sciences on the one hand; the study program is, on the other hand, shorter than that in Medicine, for example. However, students' tendency towards using the two languages was positive at both universities although it was more positive at university B than that at university A.

Finally, students' year of study was thought to be a significant factor that may drive a student to prefer a language over another in medical instruction at the university level. Respondents were sorted into five main categories (e.g., freshmen, sophomores, juniors, seniors (fourth-year students), and super seniors (fifth- or sixth-year students)). The largest proportion of respondents came from third-year students at both universities while the least number came from super senior students at university A (18 out of 302) and freshmen at university B (36 out of 302). Eventually, no significant observations had been detected in these students' perspectives towards the language of medical instruction at their universities. The results are shown in Tables 1 and 2.

The results in Table 1 show that medical students' attitudes towards using the Arabic language were positive as the mean squares were 3.09 for university A and 3.29 for university B based on the Likert scale. However, the evaluation of university B students was a little bit higher than that of university A (0.2). The same is true about using the English language as a means of medical education as shown in Table 2. The students' attitudes toward English were also positive as the mean scores were 3.08 at university A and 3.32 at university B.

It had been noticed that when the demographic variables were analyzed individually, no significant differences were identified or observed; nevertheless, when these variables interacted, differences had been identified. To ensure whether these differences were statistically significant, the researchers used factorial ANOVA as shown in Table 3.

Using the quantitative method of research, the study results showed that there were no statistical differences for all the demographic variables individually; however, there were statistical differences when the student's specific major interacted with his/her year of study only. Senior students and super senior ones who study Pure Medicine showed more positive attitudes towards the English language rather than Arabic. This could be attributed to the fact that they had a solid command of the English language; they earned high marks at schools mainly the high school; most of them attended private schools or foreign ones that teach all school subjects in English; a large group of them attended language training courses; and, finally, they had been accustomed to using Medical English after being taught in the language for three to four years at the university. The study results also showed that senior and super senior male students who study medicine showed more positive attitudes towards English as they had stronger characters and greater self-confidence or self-esteem in expressing themselves in a foreign language. They also had greater opportunities to practice English outside the campus as they could travel freely and exchange experience with others more than their female counterparts.

### 3.2. Qualitative Findings

To answer the second question that required students to provide information about their perspectives on the language of instruction in the Faculty of Medicine at the two intended universities, 55 students from each university were randomly selected, and their responses were analyzed using MAXQDA; the interviews were held in Arabic. The researcher noticed the students are divided somehow equally for their preferences in the language of instruction at the Faculty of Medicine; almost half of them preferred their mother tongue to be the medium for medical education at the university level, especially those who major in Nursing and Midwifery or Applied and Allied Medical Sciences irrespective of their gender or year of study. The other half, namely, those who study Medicine or Pharmacy, did not mind using English as a medium for medical instruction because these students were classified as good at English and their total averages in high school were high. After analyzing the students' answers using MAXQDA, the researcher sorted and categorized students' responses into four main pools or categories: advantages of using Arabic, disadvantages of using Arabic, the advantages of using English, and finally, the disadvantages of using English.

#### 3.2.1. Advantages of Using Arabic

As shown in the quantitative analysis, almost half of the students preferred their mother tongue to be the medium for medical education at the university level, especially those who major in nursing and health science irrespective of their gender or year of study. They reckon that using Arabic has more pros than cons. For instance, some respondents have stated that “the use of the Arabic language encourages students to participate in discussions in the classroom”; others have postulated that using the Arabic language “will improve academic performance and get better grades.” Furthermore, a good portion of participants has agreed that the use of the Arabic language helps to “harmonize the students' thinking and speaking” because it helps reduce the tension resulting from the use of a foreign language and thus increases the understanding of the teaching material and the content of lectures and discussions. Many students have stressed the fact that the use of the Arabic language helps to better “understand and assimilate patients,” which saves time and effort after graduation. In other words, the use of the Arabic language would “improve the quality of medical care after graduation.”

#### 3.2.2. Disadvantages of Using Arabic

Opponents of using Arabic as a medium of medical instruction believe that this will lead to a “strange Arabic language with difficult terminology” which will be different from the daily language of patients. Modern Standard Arabic could be hard enough for medical students and instructors as well; it is, to a great extent, “different from the local dialect” used in Palestine. Furthermore, the majority of staff members in medical schools have received education in other languages, mostly English, abroad. It will be difficult for them to teach in Standard Arabic, Modern Standard Arabic, and colloquial Arabic. Besides, teaching medicine in Arabic will hinder scientific development and will lead to global isolation; it will negatively affect the medical and scientific levels of students in international forums. Some participants argued that “it is not fine to use Arabic in classes and discussions and use English in exams and project discussions.” Both universities use English in exams.

#### 3.2.3. Advantages of Using English

Around half of the students who were asked to express their stance on using the medium of medical instruction preferred English over Arabic for many considerations. A large number of students stated that medical graduates who have studied in English could have better access to medical information; they made less educational effort due to the availability of educational references and resources. Others envisaged that “medical graduates who studied in English might have a higher social status in the community” since English is the first global language. Furthermore, studying medicine and health sciences in English could “provide more job opportunities and make it easier to pursue further training globally, participate in or compete for scholarships and internships or participate in international conferences.” Finally, some respondents highlighted the fact that medical graduates who have studied in English could “have better chances of working especially in universities or international medical centers,” namely, in Gulf States such as the UAE and the KSA, as job opportunities in Palestine are very few.

#### 3.2.4. Disadvantages of Using English

Teaching medicine in English is not always special or beneficial; there are some drawbacks associated with adopting the English language in the Palestinian educational context where English is used as a foreign language. It is worth noting that the use of English “discourages students from participating in discussions in the classroom,” and consequently, negatively affects academic performance and obtaining better grades. In other words, “it increases tension during exams and leads to a feeling of frustration” among students who have low English language skills and competencies. Other opponents of adopting English stated that the use of the English language “requires more effort, more time, and perhaps a greater financial burden.” Many interviewees postulated that the use of the English language may lead to “difficulty in dealing and communicating with patients after graduation” mainly because all medical graduates are expected to handle patients whose mother tongue is Arabic if they serve in Palestinian medical centers, hospitals, and clinics.

## 4. Discussion

When students were asked to justify their preferences for Arabic, many students stated that as long as they are students being taught in their mother tongue, they could understand lectures fully and adequately, increase their learning outcomes, and score better marks [5] as they tend to be more willing to participate in discussions and forums [8] because using Arabic “facilitates the process of acquiring knowledge and utilizes the optimum potential of the students” ([7], p. 562); they could express themselves better in the mother tongue [12, 13, 16]. Upon graduation, using Arabic in the workplace would help better understand the patients' presenting complaints, and consequently, improve the quality of care as well as the health status of the society [17]. The medical practitioner, namely, the doctor, will be more capable of explaining the condition to the patient and eventually spread awareness to the community.

Some students argued that using the mother tongue in medical education promotes communication skills [8] and enhances their ability to handle their patients and deal with them appropriately [34]. Teaching medical students in Arabic was very likely to create a kind of consistency or “harmony between students' thinking and speaking” (p. 1267) as well as the way they express themselves [6, 13] and will not have difficulties in communication with instructors, peers, and patients upon graduation as stated by Nilas et al. [39] who stated that “Danish doctors had difficulties in all forms of communication in English.” A gap in clinical communication may develop in countries where the native language is different from the language of medical education [40]. Some students stressed the fact that some courses (e.g., Public Health and Medical Ethics) are supposed to be taught in students' mother tongue [12]. Many languages (e.g., Arabic, French, and Hindi) have the potential to encompass all the words and expressions in all sciences including medicine; as argued by Gupta [41], any new vocabulary—even medical terms—can be adapted to Modern Standard Hindi. Medicine is taught comprehensively in Arabic in Syrian universities mainly Damascus University [2, 4].

On the other hand, some students believed that teaching medicine in Arabic is controversial and showed hesitance towards adopting as it a medium of medical education. For instance, some students in the two universities agreed that if translated medical textbooks are available in Arabic, it would be possible to teach medicine in Arabic. Moreover, about 55% of students' responses stressed the fact that Arabization will hinder scientific development and will result in isolation as all scientific gatherings, key reference books, and scientific journals use the English language and the “domestic, internationally distributed journals of medicine are often published in English” ([24], p. 284). Arabization of medical education could be a huge obstacle to students who think of pursuing education or getting employed international; future development of students who learned medicine in Arabic might decrease to the minimum, and their chances to compete in international arenas might diminish as competitions and much of the scientific, technological, and “academic information in the world is expressed in English” ([6], p. 1264).

Analyzing students' responses also showed that using English in medical education was advantageous in many aspects. For example, a lot of students indicated that medical students would have better chances to access medical information [22], “get better job opportunities” ([7], p. 562), and increase their social status, self-confidence, and self-esteem [22]. These students and graduates are more likely to participate in international competitions, sit for international exams, and be members of notable medical associations and organizations simply because “attending international conferences, courses or clerkship abroad need to be proficient in the English language” ([6], p.1264). English may be seen as an asset to one's career as stated by 96.2% of Kazakhstani medical students Kuzembayeva & Zhakanova [42]; therefore, more calls for using English to teach medicine are seen in many European countries including France [43]. Finally, some students argued that using English may reduce students' efforts, time, and money [6].

Nevertheless, teaching medicine in English is not always special or beneficial; there are some drawbacks associated with adopting the English language in the Palestinian scenario where English is used as a foreign language. For instance, students from the two universities agreed that might face difficulties in understanding concepts fully and adequately; they could not analyze certain concepts and terminologies. A lot of students show much reluctance and hesitance in class participation as they feel shy and they cannot clarify doubts; above all, they sometimes cannot express themselves freely in discussions and also on exams, especially when answering open questions, namely, for “Asian medical students who consider such open discussions as risk-taking ventures for fear of displaying ignorance, language incompetence, illogical thinking” ([44], p. 217). A large number of the respondents stated that using a foreign language is very likely to reduce motivation as students “are unable to establish an impressive academic record” (p. 133) as well as confidence levels among students as they always think about grades and new term acquisition [26]. Very few students stated that medical students may academically perform poorly [27–29] and have difficulty in communicating with patients upon graduation [30, 32, 33, 35]. Furthermore, students might not process the input presented in a second or a foreign language the way they do when mother tongues are used; they “find it too demanding and not be able to fully comprehend the academic input” ([30], p. 21). It was found that studying medicine in a foreign language may hinder students' ability to remember course details [45] in some Asian countries including Japan [46].

In conclusion, medical students' perception of the language of medical instruction shows significant disparity and discrepancy; students are divided into two categories or groups: those who prefer the mother tongue in medical education and those who see no harm in using a foreign language such as English mainly because, according to them, it is the first international language in almost all field especial sciences. Consequently, a mixture of a mother and a foreign language could be a suitable compromise in a country like Palestine. Based on the study findings, Arabic is to be used at a considerable amount so that medical students understand medicine satisfactorily and communicate with their clients upon graduation. English could be an asset, namely, to those who have future dreams outside the boundaries of their country such as international employment and participation in global gatherings and get access to modern medical advances as they are mostly introduced to people in English.

The adoption of a mixed-method technique is deemed significant in this research as it helps understand medicine students' various attitudes or perspectives towards the language of medical instruction at the university level. The researcher could analyze, interpret, and explain the quantitative results better and the qualitative ones in more detail as the study findings are mostly based on respondents' experiences and behaviors. However, it was difficult to carry out as it entailed careful selection of study sample, timing, and, on top of all, data integration in the analysis section. Furthermore, this study was carried out in two Palestinian universities; it could be better if other universities or university staff members, as well as decision-makers, were involved. Added to this, of course, was limited access to students in the other university which is located in a place that was not easy for the researcher to access without having a permit from the Israeli occupation. The small number of participants involved in the qualitative part was attributed to this fact. Time allocated to respond to the online questionnaire played a negative role and resulted in having a small, unrepresentative sample of participants who completed the survey. Therefore, a more longitudinal future study may result in different findings.

## Figures and Tables

**Table 1:** Demographic features of respondents and results related to using Arabic as a medium of medical instruction.

Variable	University A			University B		
	Number	Mean	Standard deviation	Number	Mean	Standard deviation
Gender						
Male	102	3.20	0.95	92	3.40	0.58
Female	200	3.17	0.73	210	3.21	0.54
Specific major						
Medicine	46	2.89	0.85	40	3.35	0.77
Pharmacy	114	3.45	0.70	120	3.35	0.58
Nursing and Midwifery	114	3.05	0.80	122	3.21	0.45
Applied and Allied Medical Sciences	28	3.09	0.56	20	3.29	0.39
Year of study						
Freshman (1st year)	34	2.86	1.15	36	3.27	0.58
Sophomore (2nd year)	70	3.25	0.55	70	3.38	0.52
Junior (3rd year)	112	3.17	0.74	78	3.32	0.66
Senior (4th year)	68	3.15	0.67	64	3.19	0.55
Super senior (5th or 6th year)	18	3.03	0.64	54	3.35	0.36
Total	302	3.09	0.75	302	3.29	0.56

**Table 2:** Demographic features of respondents and results related to using English as a medium of medical instruction.

Variable	University A			University B		
	Number	Mean	Standard deviation	Number	Mean	Standard deviation
Gender						
Male	102	3.28	0.97	92	3.44	0.60
Female	200	3.27	0.75	210	3.23	0.57
Specific major						
Medicine	46	2.99	0.86	40	3.36	0.79
Pharmacy	114	3.55	0.72	120	3.37	0.60
Nursing and Midwifery	114	3.15	0.82	122	3.23	0.47
Applied and Allied Medical Sciences	28	3.19	0.58	20	3.26	0.41
Year of study						
Freshman (1st year)	34	2.96	1.16	36	3.28	0.41
Sophomore (2nd year)	70	3.35	0.58	70	3.39	0.54
Junior (3rd year)	112	3.27	0.77	78	3.35	0.68
Senior (4th year)	68	3.25	0.68	64	3.18	0.57
Super senior (5th or 6th year)	18	3.13	0.66	54	3.37	0.38
Total	302	3.08	0.78	302	3.32	0.58

**Table 3:** Tests of between-subject effects (factorial ANOVA) for demographic variables.

Source	SS	DF	Mean square	*F*	*P*	*η* ^2^
University	1.36	1	1.35	3.78	0.06	0.02
Gender	1.08	1	1.09	3.07	0.08	0.01
Specific major	0.05	3	0.01	0.04	0.78	0.00
Year of study	0.57	4	0.14	0.41	0.80	0.01
University ∗ gender	0.16	1	0.15	0.43	0.51	0.00
University ∗ specific major	0.64	3	0.21	0.59	0.62	0.01
University ∗ year of study	0.88	4	0.22	0.63	0.64	0.01
Gender ∗ specific major	1.63	3	0.54	1.51	0.21	0.02
Gender ∗ year of study	3.72	4	0.93	2.62	0.22	0.04
Specific major ∗ year of study	5.41	12	0.45	1.27	0.04	0.06
University ∗ gender ∗ specific major	0.07	3	0.02	0.06	0.98	0.00
University ∗ gender ∗ year of study	0.73	4	0.18	0.51	0.73	0.01
University ∗ specific major ∗ year of study	5.35	10	1.03	1.48	0.20	0.02
Gender ∗ specific major ∗ year of study	4.66	9	0.52	1.45	0.04	0.05
University ∗ gender ∗ specific major ∗ year of study	0.57	2	0.27	0.76	0.47	0.01
Error	83.52	237	0.38			
Corrected total	118.49	601				

## Data Availability

Data will be available by the author upon request.

## Supplementary Materials

Supplementary Materials This is a questionnaire to survey the views of medical students towards the language of medical instruction at two Palestinian universities. This questionnaire consists of two parts: the first is for general information related to the student, and the second is for the questionnaire items.
