# Supplementary material for: Language of Medical Instruction in Palestine: A Mixed Method Approach of Students' Perceptions
Source: Biomed Res Int. 2022 Jul 27;2022:8999025. doi: 10.1155/2022/8999025 (PMC9348932; doi:10.1155/2022/8999025)
Supplement: Supplementary Materials — This is a questionnaire to survey the views of medical students towards the language of medical instruction at two Palestinian universities. This questionnaire consists of two parts: the first is for general information related to the student, and the second is for the questionnaire items. [file 8999025.f1.docx]

**A questionnaire to survey the views of medical students towards the language of instruction**

**Dear Student / Dear Student:**

**We extend you our best regards and thank you for the time you will spend in filling out this questionnaire, as your opinion will contribute to the development and improvement process for a group of universities in Palestine.**

**Please note that the data is treated with strict confidentiality, and it will show your name or any information related to you in any way.**

**Instructions for filling out the questionnaire:**

This questionnaire consists of two parts: the first is for general information related to the student, and the second is for the questionnaire items.

Answering the items requires choosing an estimate from five levels:

Strongly agree: It means that the statement is always true.

Agree: It means that the statement is often correct.

Neutral: It means total disapproval or complete disapproval.

I do not agree: It means that the statement is often incorrect.

Strongly Disagree: It means that the statement is not true at all.

**Section 1: General/Personal Information**

**A- Gender:** □ male □ female

**B- University:** □ An-Najah National University □ Al-Quds University, Jerusalem

**C- Specialization:** □ Human Medicine □ Pharmacy □ Dentistry □Nursing and Midwifery □ Applied and Supportive Medical Sciences

**D- Academic year:** □ first □ second □ third □ fourth □ fifth/sixth

| Domain | No. | Item | Level of Agreement | | | | |
| --- | --- | --- | --- | --- | --- | --- | --- |
|  |  |  | Strongly Agree | Agree | Uncertain | Disagree | Strongly disagree |
| Language of medical instruction | 1 | Arabic should be the language of instruction in the college. |  |  |  |  |  |
|  | 2 | English should be the language of instruction in the college. |  |  |  |  |  |
|  | 3 | The Arabic and English languages ​​must be used in the teaching of medicine and health sciences in the college. |  |  |  |  |  |
| Advantages of using Arabic | 4 | The use of the Arabic language encourages students to participate in discussions in the classroom. |  |  |  |  |  |
|  | 5 | Using the Arabic language will improve academic performance and get better grades. |  |  |  |  |  |
|  | 6 | The use of the Arabic language helps to harmonize the students' thinking and speaking. |  |  |  |  |  |
|  | 7 | The use of the Arabic language reduces the tension resulting from the use of a foreign language and thus increases the understanding of the teaching material and the content of lectures and discussions. |  |  |  |  |  |
|  | 8 | The use of the Arabic language helps to better understand and assimilate patients, which saves time and effort after graduation. |  |  |  |  |  |
|  | 9 | The use of the Arabic language would improve the quality of medical care after graduation. |  |  |  |  |  |
| Disadvantages of using Arabic | 10 | Teaching medicine in Arabic will lead to a strange Arabic language with difficult terminology which will be different from the daily language of patients. |  |  |  |  |  |
|  | 11 | Teaching medicine in Arabic will hinder scientific development and will lead to global isolation. |  |  |  |  |  |
|  | 12 | Teaching medicine in Arabic will negatively affect the medical and scientific level of students in international forums. |  |  |  |  |  |
| Advantages of using English | 13 | Medical graduates who have studied in English have better access to medical information. |  |  |  |  |  |
|  | 14 | Studying medicine in English provides more job opportunities and makes it easier to pursue further training globally. |  |  |  |  |  |
|  | 15 | Medical graduates who studied in English have a higher social status. |  |  |  |  |  |
|  | 16 | Medical graduates who studied in English make less educational effort due to the availability of educational references and resources. |  |  |  |  |  |
|  | 17 | Medical graduates who have studied in English can participate in or compete for scholarships and internships or participate in international conferences. |  |  |  |  |  |
|  | 18 | Medical graduates who have studied in English have better chances of working especially in universities or international medical centers. |  |  |  |  |  |
|  | 19 | The use of the English language discourages students from participating in discussions in the classroom. |  |  |  |  |  |
| Disadvantages of using English | 20 | The use of English negatively affects academic performance and obtaining better grades. |  |  |  |  |  |
|  | 21 | The use of the English language does not help the harmony between students' thinking and speaking. |  |  |  |  |  |
|  | 22 | The use of English may lead to difficulty in dealing and communicating with patients after graduation. |  |  |  |  |  |
|  | 23 | The use of the English language increases stress during exams and leads to a feeling of frustration among students. |  |  |  |  |  |
|  | 24 | Using the English language requires more effort, more time, and perhaps a greater financial burden. |  |  |  |  |  |
|  | 25 | Arabic should be the language of instruction in the college. |  |  |  |  |  |
